# Radial and ulnar medullary canal diameter in children: Anatomical limitations of elastic stable intramedullary nailing

**DOI:** 10.3389/fsurg.2022.882813

**Published:** 2022-10-28

**Authors:** Cui Wang, Yuxi Su

**Affiliations:** ^1^ Radiology Department, Chongqing Key Laboratory of Pediatrics, Ministry of Education Key Laboratory of Child Development and Disorders, National Clinical Research Center for Child Health and Disorders, China International Science and Technology Cooperation Base of Child Development and Critical Disorders, Children’s Hospital of Chongqing Medical University, Chongqing, China; ^2^ Department II of Orthopaedics, Chongqing Key Laboratory of Pediatrics, Ministry of Education Key Laboratory of Child Development and Disorders, National Clinical Research Center for Child Health and Disorders, China International Science and Technology Cooperation Base of Child Development and Critical Disorders, Children’s Hospital of Chongqing Medical University, Chongqing, China

**Keywords:** medullary canal diameter, elastic stable intramedullary nailing, radius fracture, ulnar fracture, children

## Abstract

**Introduction:**

Surgery is inevitable for children who cannot achieve the ideal reduction in forearm fractures. The biggest limitation of the elastic stable intramedullary nail (ESIN) fixation method is the diameter of the medullary canal. This study aimed to measure the medullary canal diameters of the radius and ulna at different ages in children.

**Methods:**

The forearm radiographs of 540 children were retrospectively reviewed. All background characteristics, including weight, sex, maturity of the radius and ulna, and length of the radius and ulna, were measured and recorded. Children with radius and ulnar diameters shorter than 2 mm were analyzed by statistical regression analysis by SPSS software.

**Results:**

When we set 2 mm as the minimum medullary canal diameter, our results showed that patients aged 3–12 years had radius and ulnar diameters under this limit. The regression analysis of risk factors with the 2 mm diameter limitation had significant differences based on age (*P* = 0.006) and sex (*P* = 0.033). There was no significant difference between patients based on weight (*P* = 0.056), ulnar length (*P* = 0.946), radius length (*P* = 0.503), radius maturity (*P* = 0.655), or ulnar maturity (*P* = 0.774).

**Conclusions:**

The average medullary canal diameter remained constant until 12 years of age. However, the average diameter length did not increase significantly after the age of 12 years. The incidence of medullary canal diameter shorter than 2 mm was correlated with age and sex. Our results suggest that surgeons should pay attention to the medullary diameter of the anteroposterior and lateral radiographs to determine the ESIN diameter.

## Introduction

Forearm fractures are the most common fractures in children, and diaphyseal fractures account for approximately 30% of these cases (1). Fortunately, most fractures can be treated conservatively by closed reduction or cast immobilization (2, 3). However, according to some studies, variations in immobilization methods have resulted in a high incidence (approximately 7%–39%) of redisplacement (4, 5). Furthermore, the body's ability for deformity correction varies depending on age. Open reduction and fixation are unavoidable for severe deformities when the fracture angle exceeds the body's correction ability.

There are two kinds of fixation methods under open surgery: plate or intramedullary fixation (4). Steel plates, elastic stable intramedullary nails (ESINs), or Kirschner wires (K-wires) are widely used fixation methods (6, 7). However, recent studies have shown that intramedullary nailing techniques are better than steel plates because they are minimally invasive, leave smaller scars, and are easy to remove (8, 9). Some studies have even proposed that one ESIN fixation of the ulna could affect both the radius and ulna (10). However, ESIN fixations are accompanied by complications such as skin irritation, incision infection, acute compartment syndrome, fracture displacement, or bone bending (11). Nonetheless, fixation stability is ultimately crucial for treating patients with fractures (11, 12).

The ESIN diameter is key for avoiding some of the fracture complications. For lower limb fractures (such as tibial and femoral fractures), 40% occupation of each ESIN has been reported to be optimal for fixation (12, 13). However, there are no reports on the best diameter ratio for ESINs in upper limb fractures. Some studies have shown that most surgeons were inclined to use the thickest ESIN possible (14). The diameters of the radius and ulna pose a limitation for inserting ESINs into these two bones.

Unfortunately, in our literature search, only one paper described femur growth (15) in children. Furthermore, no studies have been conducted to date to describe the radial and ulnar medullary canal diameters in children. Therefore, this study aimed to measure the medullary canal diameters of the radius and ulna in children at different ages. These measurements can aid the development of guidelines for choosing ESIN fixation preoperatively.

## Patients and methods

**Figure 1:**
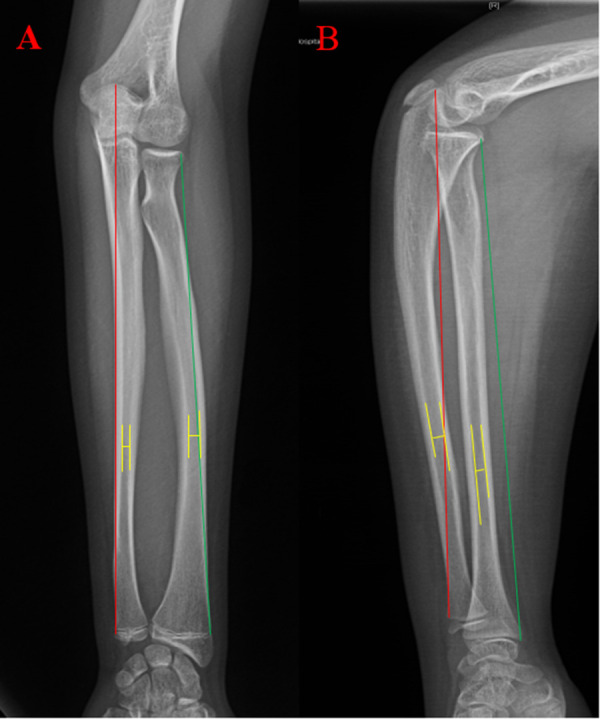
Measuring the Radius and Ulna. Measurements the length for both radius and ulna started from the proximal side to the distal side (the growth plate). The red line shows the ulnar length measurement, and the green line shows the radius length measurement. The yellow line shows the narrowest distance between the radius and ulna. The distance of the transverse line is the diameter of the radius and ulna.

Forearm radiographs from January 1, 2012, to January 1, 2020, were retrospectively reviewed at our hospital. They consisted of a consecutive series of 540 children aged 1–18 years who had forearm radiographs ordered by pediatric surgeons. The inclusion criteria are as follows: (1) normal diagnosis on forearm films and (2) patient records containing standard anteroposterior (AP) and lateral projection radiographs of forearms (Figure 1). The exclusion criteria are as follows: (1) pathological forearm bones, (2) forearm bones with fractures, and (3) nonstandard positioning of forearms in the radiographs. The ethics committee of our hospital approved this study, and all the patients' guardians provided informed consent to conduct this study.

On the AP and lateral projection radiographs of the forearms, the diaphyseal intramedullary diameters of the radius and ulna were measured at the narrowest area using GE Healthcare-Centricity RIS CE V3.0 software (General Electric Company, United States). The lengths of the radius and ulna were also measured using the same method (Figure 1). However, the wider diameter of the intramedullary canal was not measured because this study was interested in the narrower diameter for ESIN insertion. In addition, the radial and ulnar physes were evaluated using the Greulich–Pyle method (16), which was the evaluation method previously used by Luk et al. (16). The radial and ulnar maturity levels were classified into grades 11 and 9 according to the distal radial and ulnar radiographs, respectively. Grade 0 meant epiphysis had not occurred, while grades 11 and 9 of the radius and ulna, respectively, meant complete fusion with a rounded lateral/medial edge of the physeal line. Interobserver variability was assessed by the same observers repeating the same measurements and evaluations after 1 month. In addition, the patients' weight, sex, and age were also recorded.

## Statistical analysis

All statistical analyses were performed using IBM SPSS Statistics for Windows version 20.0 (IBM Corp., Armonk, New York, United States). Univariate analyses using Pearson's chi-squared and Fisher's exact tests were performed when the expected count was <5. Additionally, binary regression analysis was used to compare the outcomes based on medullary canal diameters <2 mm with age, sex, radial maturity, radial and ulnar lengths, and weight. A *P*-value <0.05 was considered statistically significant.

## Results

**Figure 2:**
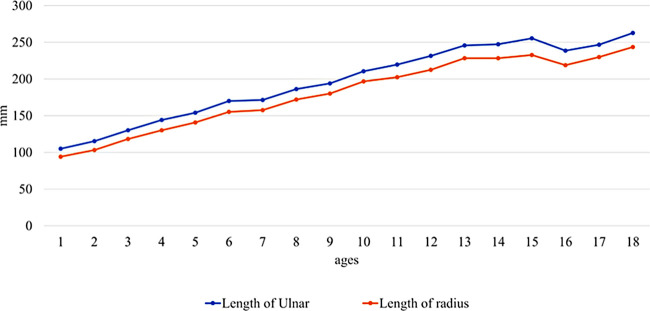
Average medullary canal diameter of radius and ulna. No significant growth was observed in either the radius or ulna between the ages of 1 and 12 years. However, starting from 13 years old, there was significant growth in the radial and ulnar diameters. The green line represents the radius, and the red line represents the ulna.

After applying inclusion and exclusion criteria, 540 patients (266 men and 274 women) were included in the analyses. There were 1,080 radiographs measured in total. Each age group had 30 patients, and the same radiologist evaluated the radiographs. The average radial and ulnar diameters are shown in Table 1 and Figure 2. Surprisingly, there was no significant difference in the average radial and ulnar diameters between the age groups (*P *= 0.722).

**Table 1:** Distribution of the medullary canal diameter from 1 to 18 years old.

Age	Ulnar MCD (mm)								Radius MCD (mm)							
	<1.0	1.0–1.5	1.5–2.0	2.0–2.5	2.5–3.0	3.0–3.5	3.5–4.0	>4.0	<1.0	1.0–1.5	1.5–2.0	2.0–2.5	2.5–3.0	3.0–3.5	3.5–4.0	>4.0
1	0	0	4	2	14	8	1	1	0	0	0	9	7	11	1	2
2	0	1	3	7	11	7	2	0	0	1	2	5	13	4	5	0
3	0	1	2	6	11	8	1	1	0	0	5	7	7	8	2	1
4	0	1	7	8	7	5	2	0	0	0	6	9	11	4	0	1
5	0	0	6	6	11	7	0	0	0	0	7	8	8	5	2	0
6	0	0	5	11	6	6	1	1	0	0	5	9	8	6	1	1
7	0	0	6	9	6	7	2	0	0	2	2	10	8	5	1	2
8	0	0	3	12	6	7	1	1	0	0	2	10	8	5	2	3
9	0	0	7	9	10	3	1	0	0	0	3	12	7	4	3	1
10	0	2	3	12	6	5	1	1	0	0	6	9	3	6	4	2
11	0	0	3	7	8	5	4	3	0	0	3	3	8	7	6	3
12	0	1	2	6	11	3	3	2	0	0	2	9	8	5	2	4
13	0	0	0	1	1	12	7	9	0	0	0	1	6	9	7	7
14	0	1	1	2	6	7	11	2	0	0	0	3	13	5	6	3
15	0	0	1	2	2	3	6	16	0	0	1	2	3	4	8	12
16	0	0	0	1	3	2	8	16	0	0	0	0	3	3	11	13
17	0	0	0	0	4	1	11	14	0	0	0	0	2	5	9	14
18	0	0	0	0	1	8	9	12	0	0	0	0	0	5	7	18

MCD, medullary canal diameter.

**Figure 3:**
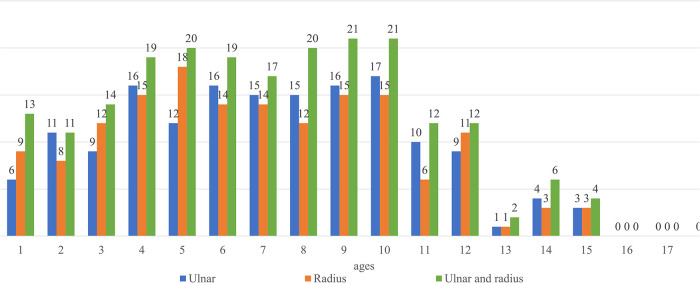
Growth of radial and ulnar lengths. The average length growth of the ulna and radius correlated with age <15 years. After 15 years, the length of the ulna and radius remained the same.

**Figure 4:**
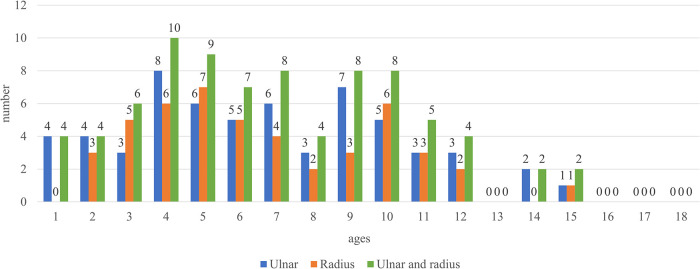
Number of children with medullary canal diameters <2.55 mm. There were >10 children in each group <13 years old.

**Figure 5:**
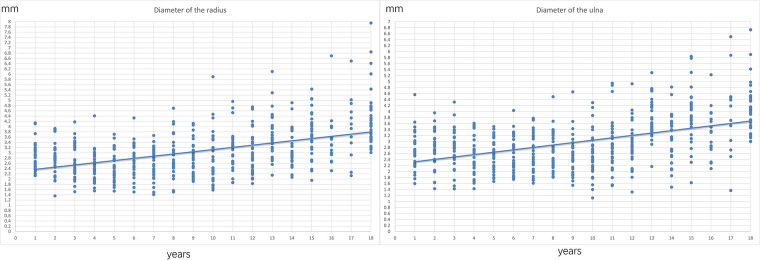
Number of children with medullary canal diameters <2 mm. Most patients with <2 mm medullary canal diameters were between the ages of 3 and 11 years.

Furthermore, radial and ulnar length growth was steady (Figure 3), with no obvious stagnation observed in diameter changes (Figure 2). When 2.5 mm was set as the minimum medullary canal diameter, the results showed that patients aged between 3 and 12 years were within this limit (Figure 4). Similarly, when 2 mm was set as the minimum diameter, the results also showed that most of the patients who fit this criterion were between 3 and 12 years old. Only the number of participants in the 8-year-old age group within the new limit significantly decreased (Figure 5). The regression analysis of risk factors with the 2-mm-diameter limit showed significant differences based on age (*P *= 0.006) and sex (*P *= 0.033). No significant difference was seen between patients based on weight (*P *= 0.056), ulnar length (*P *= 0.946), radial length (*P *= 0.503), radial maturity (*P *= 0.655), or ulnar maturity (*P *= 0.774).

## Discussion

This study focused on the medullary canal diameter variations of 1–18-year-old patients in our clinical center. Lascombes et al. reported that the medullary canal diameter significantly influences the choice of a suitable ESIN (12). Furthermore, they demonstrated that 80% of the total medullary canal diameter owed its stability to fixation (12). However, Shaha et al. questioned the 60%–80% result; nonetheless, they obtained similar outcomes for treating femoral fractures in children (17). In our clinical center, the 80% principle was applied to select the appropriate ESIN. However, forearm fractures differ from lower limb fractures in the femur and tibia, probably because the humerus does not need to bear weight.

It is also worth noting that the radius and ulna only require one ESIN for fixation; however, the key issue is how the ESIN diameter is chosen. Most pediatric surgeons choose ESINs with 2 mm, 2.5 mm, or even larger diameters. However, we recommend that surgeons should measure the canal diameter preoperatively to ensure proper nail placement in the canal and avoid surgical mistakes. For instance, if the selected ESIN diameter exceeds the actual diameter of the forearm, it may cause difficulties in inserting the ESIN intraoperatively and may also make it hard to remove the nail when the fracture heals. Hence, this measurement can assist surgeons in choosing the right ESIN size.

The present study demonstrated that children aged between 2 and 13 years were highly likely to have medullary canal diameters <2 mm on AP and lateral radiographs. Furthermore, it is known that the nails could be incarcerated or even cause iatrogenic fractures if too much force is applied during nail insertion in these patients (18). This study also showed that the medullary canal diameter size remained constant in the forearms of children aged 3 to 12 years. Additionally, there was no significant growth of the medullary canal diameter between the ages of 1 and 12 years. However, significant enlargement was observed for both radial and ulnar medullary canal diameters after the age of 13 years.

To our knowledge, there are limited studies on medullary canal diameter measurements (15). Lucak et al. retrospectively analyzed femurs in children aged 1 to 10 years, and they found a strong correlation with age and observed no limitations with inserting 2.5-mm-diameter ESINs. However, in the present study, there was no strong correlation with age, and 2-mm-diameter ESINs could not be used in some children, particularly those aged between 4 and 12 years. In addition, this study's results showed that several children had radial or ulnar diameters <2 mm (Figure 5). Consequently, other fixation materials, such as K-wires, could be better for these patients.

K-wires, steel plates, and ESINs are the most common and widely used fixation materials for forearm fractures in children (7, 19–21). Emerging studies increasingly recommend ESIN as the first choice for fixation due to its associated low complication rate and economic feasibility (7, 19). Our clinical center uses ESINs as the first choice when closed reduction and fixation by cast fail to fix the bone. Lindley et al. have stressed that improvements in the ESIN fixation technique could eliminate several complications related to the technique; for example, cutting the bent tip could facilitate nail passage (18). Johnson et al. have demonstrated that there is no need for ESINs to be fully inserted. Three times of the length of the bone's diameter that passed the fracture line could provide enough stability (13). In addition, Jeffrey et al. proposed that a single ESIN ulna fixation could be a safe and efficacious method (10). These studies all provide reports that can decrease the difficulty of ESIN fixation.

Furthermore, while most studies used 2-mm, 2.5-mm, and 3-mm-diameter ESINs for the fixation of forearm fractures, the present study focused on ESINs with diameters <2 mm and 2.5 mm. This study's results suggest that surgeons should pay attention to the medullary diameter on AP and lateral radiographs to determine the ESIN diameter (Figures 4, 5). Furthermore, all surgeons should consider the medullary canal's shape important. Because the medullary canal does not have a regular or smooth shape, the insertion of the ESIN may be more complicated when only the diameter of the ESIN nail is considered.

This study attempted to determine the risk factors associated with having medullary canal diameters <2 mm; the results showed a high correlation of medullary canal diameters with age and sex. However, there were no significant correlations between the radial and ulnar lengths, maturity of the radius or ulna, and weight and the medullary canal diameter. These results showed that the radial and ulnar diameters were quite different from that of the femur, with significant variations (22). Furthermore, diameters of <2.5 mm were uncommon after the age of 13 years; however, ESIN was less likely to be used in patients in this age group. This was because one of the indications for surgery was fractures in patients with 1–2 years of growth remaining (14). For these near-mature patients, a steel plate is an optimal option (14).

Based on this study, the authors suggest the following guidelines for choosing ESIN fixation preoperatively: (i) there may be some limitations with radial or ulnar diameters of <2 mm in patients aged 4–12 years. Hence, other fixation materials, such as K-wires, could be the better choice. However, the occurrence of this limitation did not correlate with sex or forearm length. (ii) For most patients aged 1–3 years and >12 years, ESIN fixation can be adopted safely.

This study had some limitations. First, this was a retrospective study, with all clinical data drawn from a single center; however, a prospective study may have less bias. Second, the study population for each group was small and more patients are needed in future studies. Third, data analysis was performed using x-ray radiographs only; computed tomography or cadavers would provide much more accurate measurements. Fourth, all the data were acquired from children without fractures. Finally, most of the patients were of the same ethnicity and from the same region of the country (southeast). Consequently, this study's results might not correlate with those from other ethnicities and countries; therefore, international studies involving multiethnic groups are necessary to evaluate the generalizability of our findings.

## Conclusion

ESIN is a safe technique requiring minimally invasive fixation materials and is widely used in children. However, the biggest limitation of this fixation method has been the diameter of the medullary canal. In this study, we proved that the average medullary canal diameter remained constant in the 3–12-year-old age group, and the occurrence of medullary canal diameters of <2 mm was correlated with age and sex. Hence, we recommend that surgeons pay significant attention to the medullary canal diameters observed on AP and lateral radiographs.

## Data Availability

The raw data supporting the conclusions of this article will be made available by the authors, without undue reservation.
